# Supplementary material for: Plasma-wakefield accelerator simultaneously boosts electron beam energy and brightness
Source: Nat Commun. 2025 Nov 28;16:10719. doi: 10.1038/s41467-025-65742-8 (PMC12663360; doi:10.1038/s41467-025-65742-8)
Supplement: Supplementary file 1 — Supplementary Information [file 41467_2025_65742_MOESM1_ESM.pdf]

Supplemental Information

Plasma-Wakefield Accelerator Simultaneously Boosts Electron Beam Energy and Brightness

Chaojie Zhang[1,*], Douglas Storey[2], Alexander Knetsch[2], Brendan D. O'Shea[2], Robert Ariniello[2], Gevy J. Cao[3], Sebastien Corde[4,2], Thamine N. Dalichaouch[5], Claudio Emma[2], Ole G. Finnerud[3], Spencer Gessner[2], Claire Hansel[6], Elias Hansen[5], Valentina Lee[6], Carl A. Lindstrom[3], Mike Litos[6], Nathan Majernik[2], Kenneth A. Marsh[1], Warren B. Mori[5], Ivan Rajkovic[2], Mark J. Hogan[2], and Chan Joshi[1,*]

Emails: chaojiez@ucla.edu; cjoshi@ucla.edu;

1. Department of Electrical and Computer Engineering, University of California Los Angeles, Los Angeles, California 90095, USA
2. SLAC National Accelerator Laboratory, Menlo Park, California 94025, USA
3. Department of Physics, University of Oslo, Oslo 0316, Norway
4. Laboratoire d'Optique Appliquée, ENSTA, CNRS, Ecole Polytechnique, Institut Polytechnique de Paris, 91762 Palaiseau, France
5. Department of Physics and Astronomy, University of California Los Angeles, Los Angeles, California 90095, USA
6. Department of Physics, Center for Integrated Plasma Studies, University of Colorado Boulder, Boulder, Colorado 80309, USA

Supplementary Figures

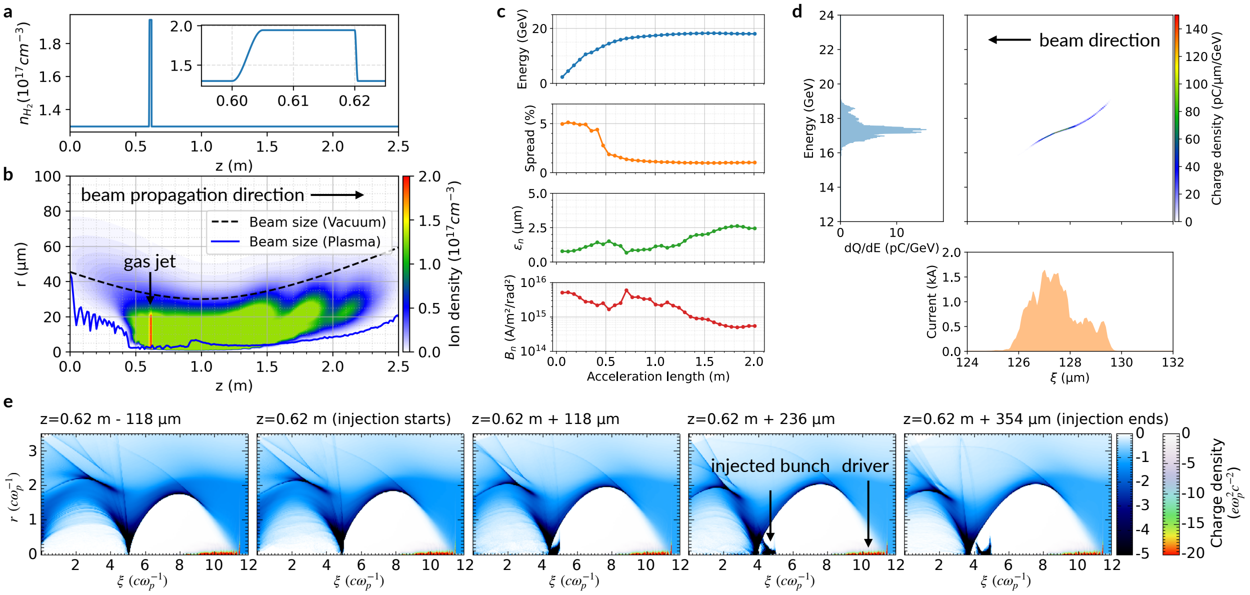

Supplementary Fig. 1 | PIC simulation. a, Plasma density profile used in the simulation. Inset shows detailed view of the gas jet region. **b,** Transverse ion density distribution as a function of propagation distance. Each column represent the ion density

extracted from one frame of the simulation, evaluated at one plasma skin depth, c/ω_p, after the ionization front in the moving window. The beam size curves clearly show that the best focus position of the electron beam (black-dashed) moves upstream (blue solid) and the beam is more tightly focused by the partially ionized plasma (see color bar of the ion density). Consequently, the gas jet and the subsequent meter-scale hydrogen gas region are fully ionized by the tightly focused electron beam. The on-axis ion density is lower because the simulation models the drive bunch with a Gaussian transverse profile, which has zero transverse electric field on-axis to cause ionization. **c,** Evolution of injected bunch parameters as a function of acceleration length: energy (blue), relative energy spread (orange), normalized transverse emittance (green), and beam brightness (red). The rapid drop in energy spread at 0.4 m is due to charge loss at the back of the injected bunch caused by wake contraction from the onset of drive bunch energy depletion. This charge loss does not affect the peak current of the bunch. **d,** Longitudinal phase space of the injected bunch after one meter of acceleration (at z=1.6 m in **b**), with energy spectrum and current profile shown in the top left and bottom panels, respectively. The LPS undergoes only minor changes through the remaining plasma length. The LPS at this location is used for benchmarking the machine learning LPS reconstruction method. **e,** Snapshots from the OSIRIS simulation showing the charge density of plasma electrons during the downramp injection process. The frames illustrate the trapping of plasma electrons into a distinct bunch at the back of the wake as the driver traverses the 500-μm-long $\sin^2$-shape density downramp, which starts at z≈0.62 m and drops from 1.5 to 1.0 n_p, where $\mathrm{n_p} \approx 1.3 \times 10^{17}$ $\mathrm{cm^{-3}}$.

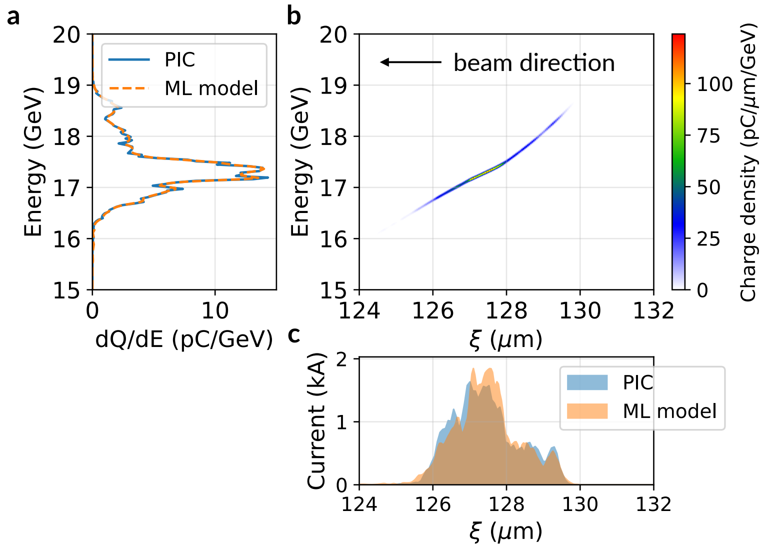

Supplementary Fig. 2 | Verification of the LPS reconstruction using PIC simulated energy spectrum. a, Comparison between PIC-simulated energy spectrum (blue line) and ML model reconstruction (orange dashed line). **b,** Reconstructed longitudinal phase space from the ML model showing the energy-position correlation of injected electrons with charge density indicated by the color scale. This LPS agrees well with the PIC-simulated result shown in Supplementary Fig. 1d. **c,** Reconstructed current profile using the ML model (orange) agrees reasonably well with the true current distribution from the PIC simulation (blue).
